# Supplementary material for: Colloidal gel elasticity arises from the packing of locally glassy clusters
Source: Nat Commun. 2019 May 20;10:2237. doi: 10.1038/s41467-019-10039-w (PMC6527676; doi:10.1038/s41467-019-10039-w)
Supplement: Supplementary file 1 — Supplementary Information [file 41467_2019_10039_MOESM1_ESM.pdf]

Supplementary Information for: Colloidal Gel Elasticity Arises From the Packing of Locally Glassy Clusters

Kathryn A. Whitaker,[1,†] Zsigmond Varga,[2] Lilian C. Hsiao,[3] Michael J. Solomon,[4] James W. Swan,[2,*] and Eric M. Furst[1,*]

[1]Department of Chemical and Biomolecular Engineering
University of Delaware, Newark, Delaware 19716
[2]Department of Chemical Engineering, Massachusetts Institute of Technology
Cambridge, MA 02139, USA
[3]Department of Chemical and Biomolecular Engineering,
North Carolina State University, Engineering Building I, Raleigh NC 27695, USA
[4]Department of Chemical Engineering, University of Michigan
Ann Arbor, Michigan 48109, USA

*Corresponding authors; E-mail: jswan@mit.edu (ORCID 0000-0002-4244-8204)
and furst@udel.edu (ORCID 0000-0002-8849-4484).
†Current address: The Dow Chemical Company, 1702 Building, Midland, Michigan 48667

Supplementary Note 1.

The complex modulus of all the colloidal gels under study was measured experimentally and computed in simulations. In experiments, a small amplitude frequency sweep was conducted using a strain controlled protocol. Sensitivity at low frequencies is lower in strain control than stress control, so deviations from the stress controlled measurement of the zero frequency modulus reported in the main text are expected particularly in the weakest gels. At higher frequencies, there is significant hydrodynamic stress generated by the imposed flow, and the torque limits of the rheometer no longer hinder the measurement. In the simulations, a swept-sine strain signal was used to deform the periodic box containing the colloidal gel in oscillatory simple shear. The shear stress was computed at each point in time, and the ratio of the Fourier transform of the stress and strain was used to define the complex modulus.

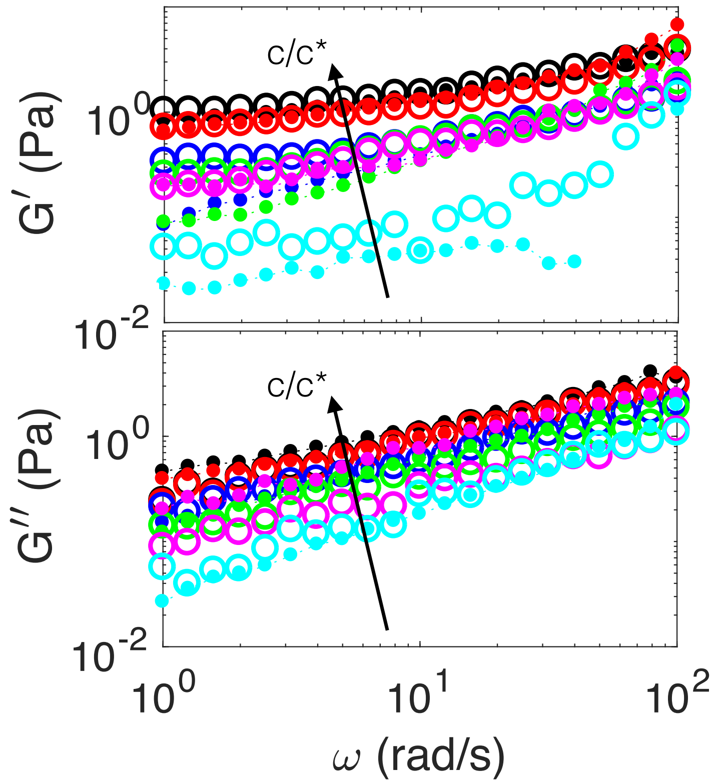

Supplementary Figure 1: Comparison of linear viscoelasticity in experimental and simulated gels. The linear viscoelasticity for the six different gels investigated experimentally and in simulations. Closed symbols are experimental measurements on a strain controlled rheometer. Open symbols are the results of dynamic simulations. The polymer concentrations correspond to symbol colors as follows $c/c^* = 0.35$ (light blue), 0.47 (magneta), 0.59 (green), 0.71 (dark blue), 0.83 (red), 0.94(black).

Supplementary Note 2.

The cluster model implicit equation

$$G' = \phi_c z_c(\phi_c)\kappa/(5\pi) \tag{1}$$

was also applied to the rheology measurements on a depletion system of octadecyl silica suspended in decalin with a polystyrene depletant reported by Ramakrishnan and coworkers. The particle and depletant sizes are an order of magnitude smaller and the particle and polymer chemistries are different. The ratio of R_g/a is comparable between our study and the work of Ramakrishnan et al. (0.06 and 0.08, respectively), such that although the particles are two orders of magnitude different in size, the relative range and magnitude of the depletion attraction is similar. Their results also include samples over a range of volume fractions.

The correlation length of these gels (ξ = 327 nm) was determined by ultra-small angle x-ray scattering (USAXS) and was also found to be nearly independent of volume fraction and attraction strength for the range of polymer concentrations $c/c^* = 0.3$–0.8. We find ϕ_g from eqn. 1 fitting the reported modulus of the gels (cf. Supplementary Figure 2). The model agrees well with the results at $c/c^* = 0.30$ and 0.60, but begins to deviate from the measurements at the lower volume fractions $c/c^* = 0.20$ and 0.15; the measured moduli are lower than the model predictions. However, since there is no structural information provided for the gels at $c/c^* = 0.20$ or 0.15, the assumption of a constant ξ at these values of c/c^* has not been established and may not be accurate. Adjusting the value of ξ from the constant value 327 nm to 730 nm for $c/c^* = 0.15$ and 456 nm for $c/c^* = 0.20$ improves the model agreement with the experimental results as shown by the dashed lines in Supplementary Figure 2.

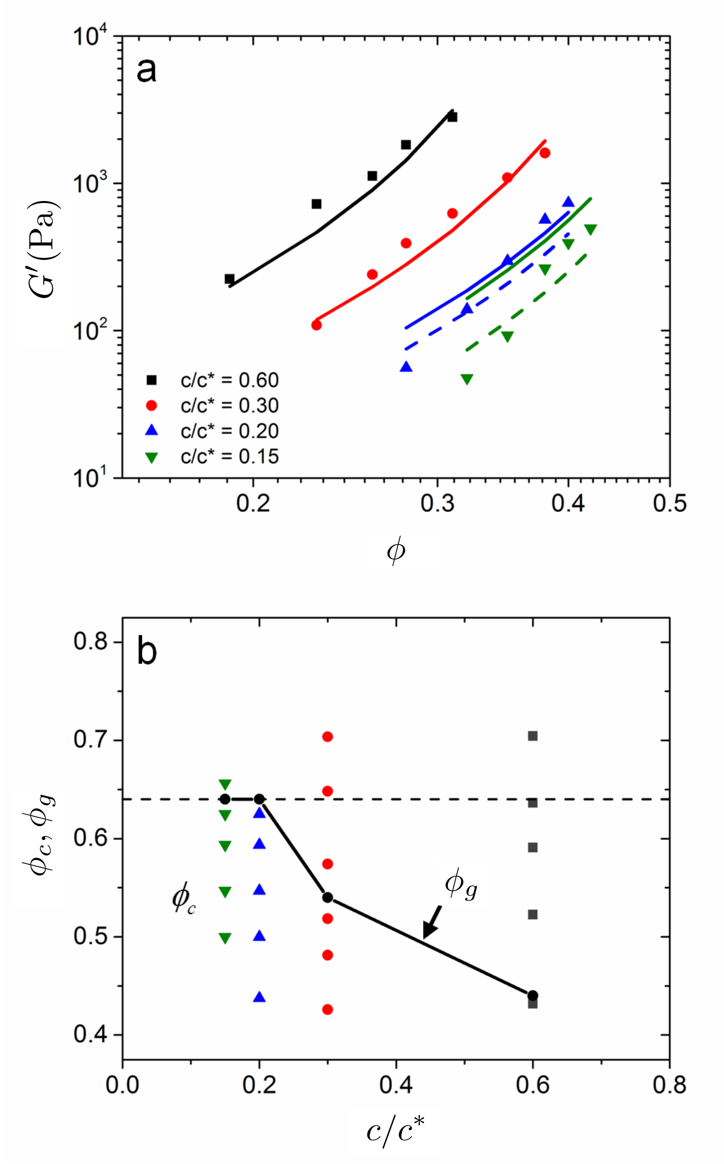

Supplementary Figure 2: Comparison to octadecyl silica depletion gels. **a** Measured elastic modulus of octadecyl silica depletion gels in decalin as a function of bulk volume fraction. The data are adapted from Ramakrishnan and co-workers (S. Ramakrishnan, Y.-L. Chen, K. S. Schweizer, and C. F. Zukoski. Elasticity and clustering in concentrated depletion gels. Phys. Rev. E, 70:040401(R), 2004.) The solid lines are G' calculated from eqn. 1 with $2a = 90$ nm, $R_g = 3.5$ nm, $\xi = 327$ nm, and $\delta = 1$ nm. The dashed lines are G' calculated with $\xi = 456$ nm at $c/c^* = 0.20$ and $\xi = 730$ nm at $c/c^* = 0.15$. **b** Cluster particle density ϕ_g values (circles connected by black line) used to calculate ϕ_c (individual data points) $\phi_g = \phi/\phi_c$ to input into the model. The data are for a range of volume fractions so that at a constant c/c^*, the cluster volume fraction ϕ_c increases with the bulk particle volume fraction ϕ.
